# Percutaneous coil embolization of a post-traumatic left anterior descending coronary artery pseudoaneurysm: a case report

**DOI:** 10.1093/ehjcr/ytae625

**Published:** 2024-11-26

**Authors:** Joaquin Espinoza, Marina Byer, Moises Vasquez, Dileep R Yavagal, Yiannis S Chatzizisis

**Affiliations:** Division of Internal Medicine, University of Miami, Leonard M. Miller School of Medicine, 1400 NW 12th Ave, Miami, FL 33136, USA; Division of Cardiovascular Medicine, University of Miami, Leonard M. Miller School of Medicine, 1400 NW 12th Ave, Miami, FL 33136, USA; Division of Internal Medicine, University of Miami, Leonard M. Miller School of Medicine, 1400 NW 12th Ave, Miami, FL 33136, USA; Division of Neurosurgery and Endovascular Neurosurgery, University of Miami, Leonard M. Miller School of Medicine, 1400 NW 12th Ave, Miami, FL 33136, USA; Division of Cardiovascular Medicine, University of Miami, Leonard M. Miller School of Medicine, 1400 NW 12th Ave, Miami, FL 33136, USA

**Keywords:** Coronary artery pseudoaneurysm, Left anterior descending artery, Case report, Cardiac trauma, Percutaneous intervention, Coil embolization

## Abstract

**Background:**

Coronary artery pseudoaneurysm (PSA) is a rare occurrence linked to percutaneous coronary interventions (PCIs), infection, or chest trauma, lacking established management guidelines due to its low incidence.

**Case summary:**

A 78-year-old male with a medical history of triple vessel disease, post coronary artery bypass grafting, heart failure, and chronic obstructive pulmonary disease, presented with intractable left-sided chest pain following a mechanical fall. The initial workup was positive for mildly elevated high-sensitivity troponin and brain natriuretic peptide raising suspicion for a pulmonary embolism; but chest computed tomography angiography revealed an enlarging pericardial haematoma. Further computed tomographic coronary angiography exposed a mid-left anterior descending (LAD) interrupted segment concerning for a contained ruptured PSA. Left heart catheterization confirmed the suspicion, showing a collection of contrast at the haematoma site following injection of contrast into the saphenous vein graft to the diagonal artery. The patient underwent percutaneous PSA coiling, successfully occluding blood inflows from both the first diagonal and distal LAD. There were no subsequent electrocardiogram changes or further elevation in troponin levels ensuring the integrity of the LAD vital branches.

**Discussion:**

Coronary PSA results from the dissection of at least one layer of the vessel wall leading to blood extravasation. Although they are usually associated with PCI complications, the absence of haemopericardium in prior imaging makes the recent blunt chest trauma the most likely cause of this patient’s presentation. Percutaneous coiling of inflow vessels to PSAs proved to be a suitable option in this case of a patient with a history of sternotomy and an expanding pericardial haematoma.

**Learning points**

- To recognize the most common risk factors and clinical presentations of coronary pseudoaneurysm and raise awareness to prevent fatal complications.
- To underscore the importance of coronary computed tomography and cardiac magnetic resonance imaging in the diagnostic process of coronary pseudoaneurysm.
- To emphasize the significance of conducting a thorough analysis of the patient's comorbidities, and coronary anatomy when determining the optimal therapeutic strategy.

## Introduction

Coronary pseudoaneurysms (PSAs) are focal dilations of a coronary artery segment resulting from the dissection of at least one layer of the vessel wall leading to blood extravasation. Unlike aneurysms, PSAs are contained by the tunica adventitia or the perivascular tissue.^1^ They are associated with percutaneous coronary interventions (PCIs), infection, or trauma; being the former the most common cause.^1,2^ Despite PSAs being found in asymptomatic patients, they commonly present as acute coronary syndrome (ACS) or stable angina. If not treated, they are likely to enlarge and rupture, causing cardiac tamponade and shock. Therefore, close monitoring and prompt treatment are required to prevent complications.^2,3^

## Summary figure

Suggested algorithm for the management of patients with coronary pseudoaneurysm.^4,5^ Modified from Zhu *et al*. and Kawsara *et al*.

**Figure:**
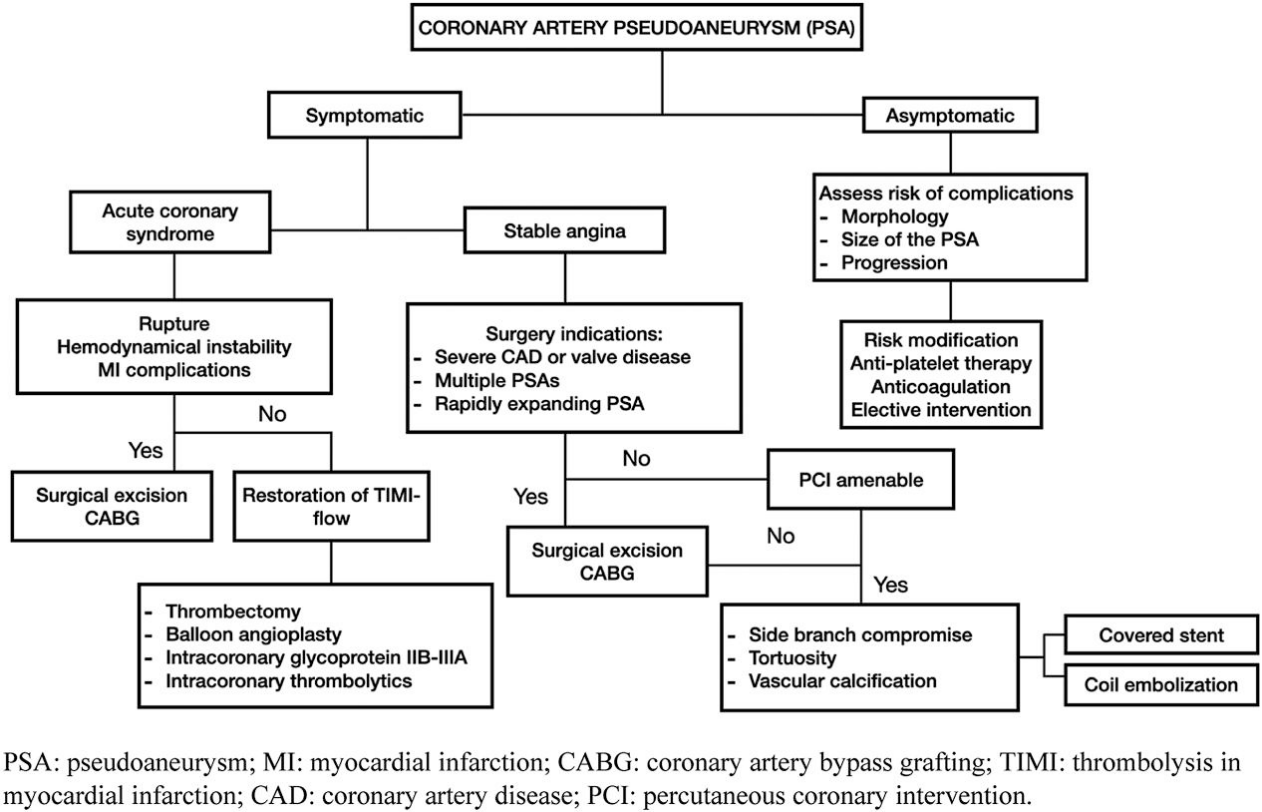


## Case presentation

A 78-year-old male with a history of triple vessel disease status post coronary artery bypass grafting (CABG) 3 years prior to presentation [left internal mammary artery (LIMA) to mid-left anterior descending artery, saphenous venous graft (SVG) to the first diagonal branch, and SVG to the second obtuse marginal], heart failure with preserved ejection fraction, and hypertension; was admitted to the hospital for left chest pain following a mechanical fall. Initial physical examination was notable for haematoma and swelling on the left chest wall, but no cardiac pain or dyspnoea was reported at presentation. Initial chest computed tomography angiography (CTA) was unremarkable except for an incidental aneurysm of the abdominal aorta.

**Figure 1:**
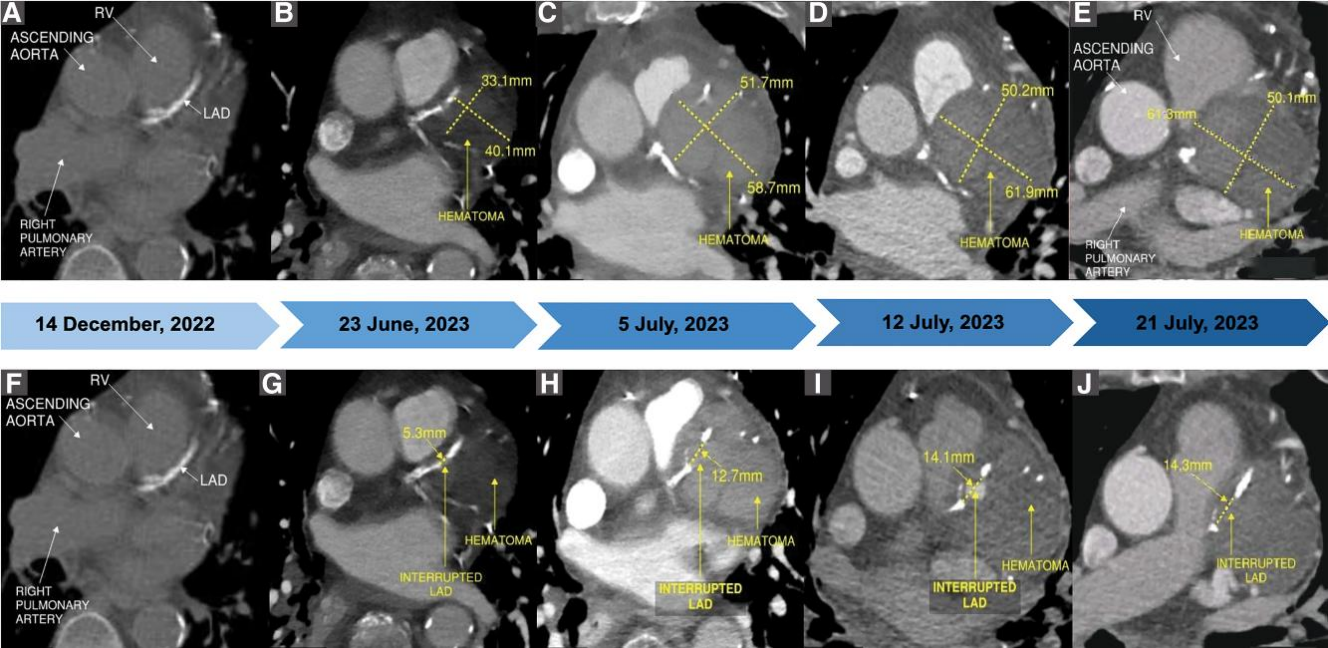
Imaging timeline: chronological imaging of pericardial haematoma growth (A–E) and LAD dissection evolution (F–J). RV, right ventricle; LAD, left anterior descending artery.

Fourteen days later, the patient reported moderate, pressure-like chest pain with dyspnoea. The patient was mildly tachycardic but no signs of hypoxia. An electrocardiogram (EKG) revealed normal sinus rhythm with frequent premature atrial complexes. Bloodwork indicated an elevated high-sensitive troponin (HsTn) level of 140 ng/mL (normal range: 0–12 ng/mL) and a brain natriuretic peptide (BNP) level of 3363 pg/mL (normal range: 0–450 pg/mL). A pulmonary embolism was suspected due to the dyspnoea. Therefore, a chest CTA was performed and, although negative for PE, it revealed a hyper-attenuating structure in the left pulmonic pericardial recess concerning for a haematoma not previously seen (*Figure 1*).

The differential diagnosis for pericardial haematoma included: left anterior descending artery (LAD) PSA, mycotic aneurysm, pericardial injury, connective tissue disorder, or malignancy with central haemorrhage. Considering recent left-sided chest trauma, imaging, and negative blood cultures, an LAD PSA was the leading possibility.

**Figure 2:**
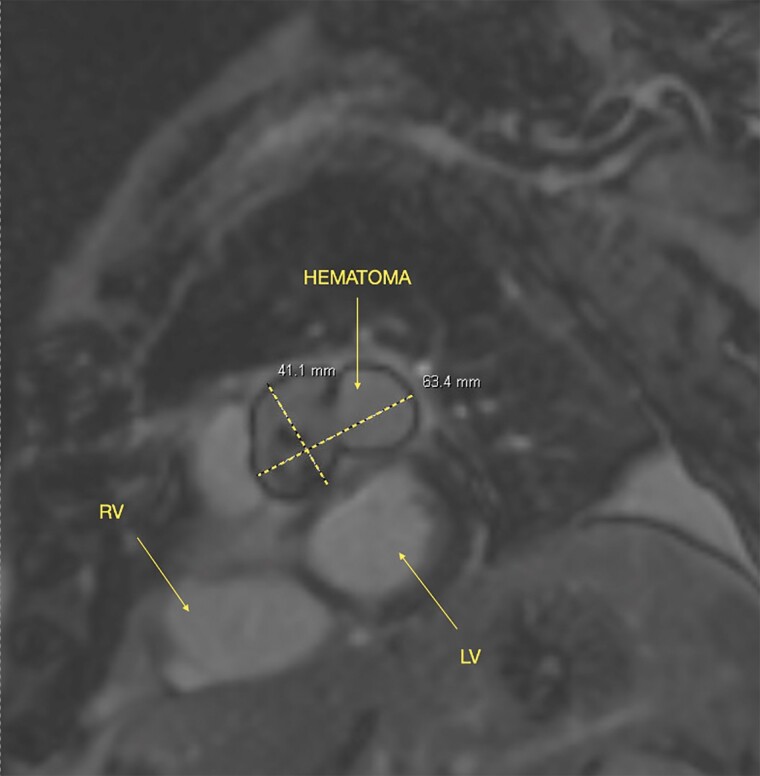
Cardiac MRI. Hematoma, haematoma; RV, right ventricle; LV, left ventricle.

Two days later, a cardiac magnetic resonance imaging (MRI) confirmed the presence of blood within the mass, validating the suspicion of a haematoma (*Figure 2*). Although the echocardiogram was unremarkable, a subsequent coronary CTA identified a 1.4 cm interrupted segment in the mid LAD (*Figure 1*), proximal to the diagonal branch take-off, with the haematoma compressing the pulmonary trunk (*Figure 1*). This finding corroborated the diagnosis of a contained ruptured PSA. Additionally, a comparative analysis of this coronary CTA with previous CTAs showed a progressive increase in the haematoma and the LAD disruption since the patient’s admission (*Figure 1*).

**Figure 3:**
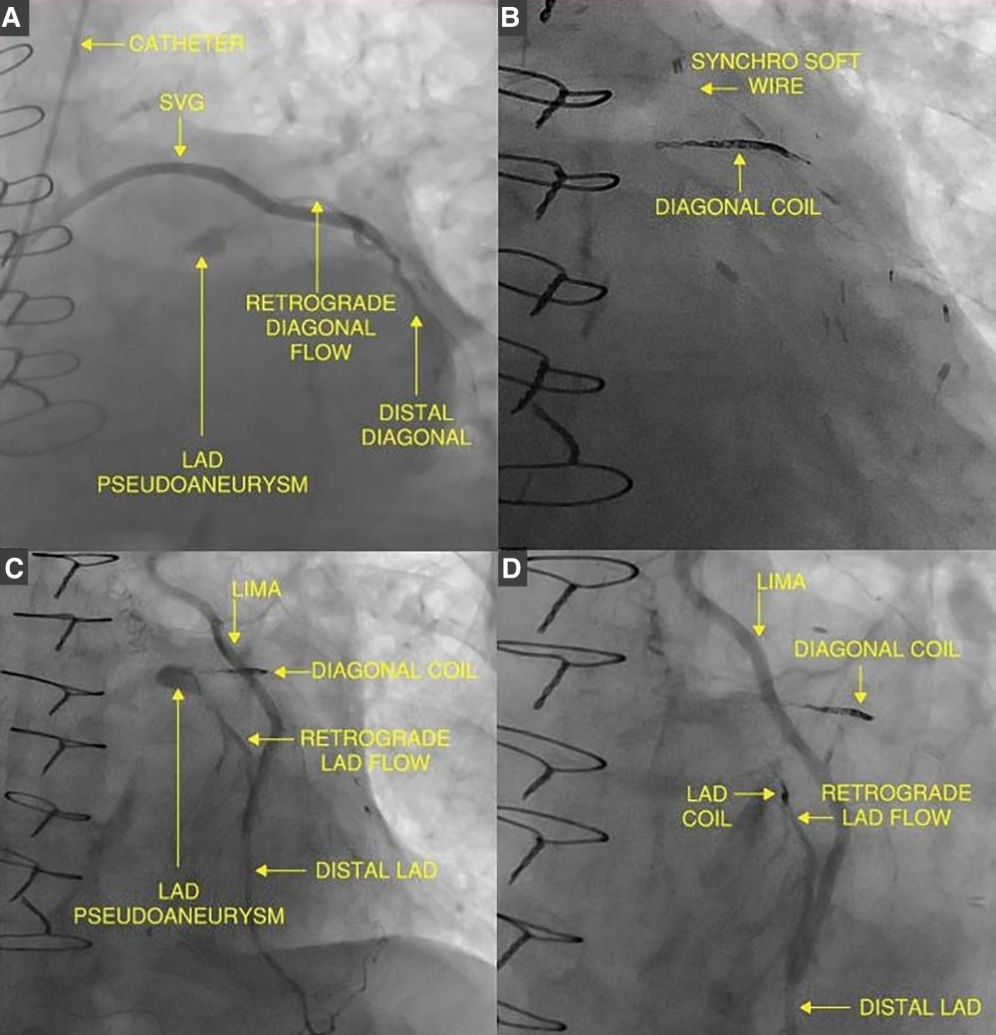
Cardiac catheterization. (A) Coronary angiogram revealing a pseudoaneurysm in the LAD. (B–D) Images from percutaneous intervention, providing insights into the procedural steps. SVG, saphenous venous graft; LAD, left anterior descending artery; LIMA, left internal mammary artery.

The following day, the patient underwent left heart catheterization, which revealed a collection at the proximal LAD after injecting contrast into the SVG directed at the diagonal artery (*Figure 3A*). In contrast, no collection was observed with LIMA injection. Aside from the patient’s existing proximal LAD chronic total occlusion (CTO), no additional coronary obstructions were found. These findings aligned with previous imaging confirming a ruptured PSA.

A multidisciplinary discussion was held to evaluate the best approach. Contemplating the patient’s presentation, comorbidities, and previous sternotomy, cardiology opted for a percutaneous PSA coiling. Considering the CTO of the proximal LAD, the plan was to use the SVG to access the diagonal branch and coil the segment just below the PSA, blocking its blood inflow.

Using a left ulnar artery access, we engaged the SVG leading to the diagonal artery. With an Excelsior SL-10 microcatheter (Stryker), we advanced a synchro soft wire, positioning it distal to the PSA in the LAD. Then, a Target 360 Nano coil (Stryker) was deployed in the proximal first diagonal vessel, just distal to the PSA. Subsequently, three additional coils were deployed in the same area resulting in the complete interruption of the PSA’s inflow (*Figure 3B*).

Surprisingly, further angiography after coiling showed new retrograde flow into the PSA from the LAD via the LIMA (*Figure 3C*). Consequently, the LIMA graft was addressed using a Vista Brite Tip Guiding Catheter (Cordis). The PILOT 50 wire (Abbott), advanced over a SuperCross 120 microcatheter (Teleflex), was utilized to access the LAD through the LIMA. Finally, we deployed a Penumbra coil to the LAD just distal to the PSA. The final angiographic images depicted good flow in the LIMA and SVG to the diagonal graft with no PSA filling (*Figure 3D*). There was no compromise to the LAD or diagonal vital branches, and careful attention was given to preserve flow across all septal perforators. No EKG changes or elevation in troponin levels were observed. Following the procedure, aspirin monotherapy was continued.

A one-week-control CTA coronary revealed a stable haematoma with no PSA filling (*Figure 1*). The patient was discharged to rehabilitation in preparation for his abdominal aortic PSA repair. One month later, the patient reported doing well at a surgery evaluation. Unfortunately, three months after the initial presentation, days before his cardiology follow-up appointment, we received news of the patient’s unexpected demise. No autopsy was conducted to determine the cause. Given the patient’s completion of rehabilitation and reports indicating no cardiac symptoms, it is suspected that his death may have resulted from a complication related to the abdominal PSA.

## Discussion

Here, we present a case of challenging diagnosis and treatment of a ruptured left anterior descending artery PSA managed with percutaneous coil embolization.

In general, PSAs are not common findings, and they are usually associated with prior coronary intervention. In this case, although the patient’s extensive cardiac history might have contributed to his presentation, it is unlikely that the PSA was caused by previous medical procedures. The absence of haemopericardium in imaging from prior hospitalizations makes fall and chest trauma the most likely mechanism. However, it is uncertain whether the force of fall or the trauma’s location triggered the following events.

It is interesting that the patient only had mild symptoms and a minimal HsTn elevation despite having a ruptured PSA and a growing pericardial haematoma. These cases are associated with a pericardial mass of blood stasis due to a slower rising of coagulated blood rather than continuous bleeding from the damaged vessel.^6^ As noted in the coronary angiography, there was no contrast leaking, and the haematoma was contained by the pericardium. Additionally, the presence of a haematoma with a narrow neck supported our diagnosis of a contained ruptured PSA, as opposed to a wide-necked lesion, which is more characteristic of a true aneurysm.

Due to its rare occurrence, there are no guidelines for the management of PSA. Following the summary figure provided, while medical therapy has been described for stable cases, symptomatic PSAs warrant a surgical approach.^4,5^ However, given that the patient was not a surgical candidate due to his prior history of sternotomy and multiple comorbidities, the medical team discussed different percutaneous options, including isolating the PSA, coiling the PSA itself, or placing a stent at the LIMA and SVG touchdown.^4,5,7^

**Figure 4:**
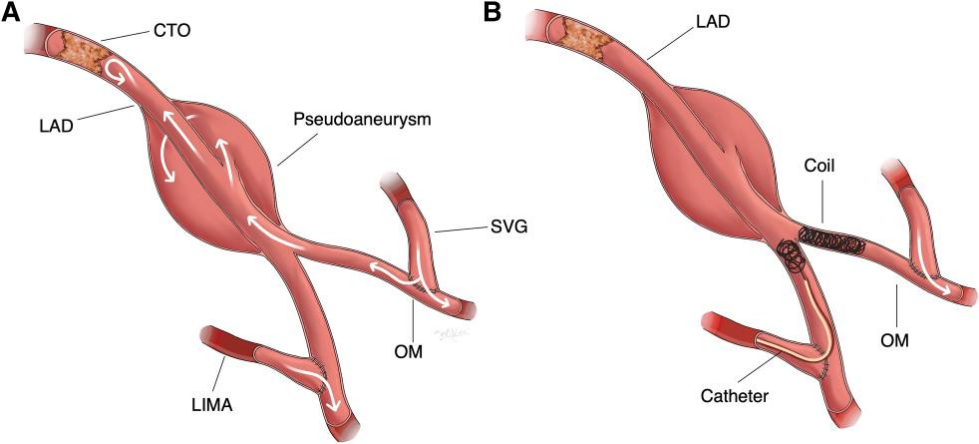
Illustrative drawing depicting key anatomical structures and the procedural steps involved in the intervention. CTO, chronic total occlusion; LAD, left anterior descending artery; LIMA, left internal mammary artery; OM, obtuse marginal; SVG, saphenous vein graft.

In this case, several challenges needed to be addressed: accessing the PSA through graft veins, preserving vital branches (including septal perforators), and the coiling of a ruptured PSA in the presence of a continuously expanding haematoma. After a thorough assessment, we isolated the PSA by coiling the retrograde inflows adjacent to the lesion as it presented fewer chances of obstructing collaterals and reduced the risk of exacerbating the PSA rupture (*Figure 4*). The absence of post-procedural EKG changes or troponin elevation indicated no procedural iatrogenic ischaemia.

## Conclusion

Coronary PSA is a complication of PCI and chest trauma. Common presentations are cardiac tamponade and ACS. Coil embolization of inflow vessels is a good option for patients with multiple comorbidities and history of sternotomy.

## Lead author biography


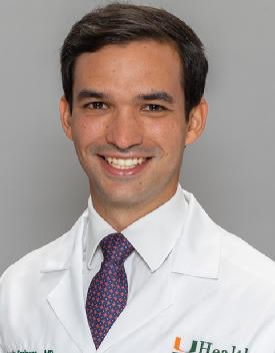


Joaquin Espinoza is currently a PGY-3 Internal Medicine Resident at the University of Miami Hospital and Jackson Memorial Hospital in Miami, Florida. He earned his medical degree at the Universidad Central de Venezuela in Caracas. Joaquin's primary clinical interest lies in becoming an Interventional Cardiologist.

## Data Availability

The data underlying this article cannot be shared publicly due to privacy concerns for the individuals who participated in the study. The data will be made available upon reasonable request to the corresponding author.

## Notes

**Consent:** Informed consent was obtained from the patient for the publication of this case report and any accompanying images. The patient was unable to review the final manuscript due to their passing. All identifying details have been anonymized to protect the patient's privacy, with particular attention placed on ensuring the highest standards of confidentiality. This consent process is compliant with COPE guidelines.

**Funding:** This article received no specific grant from any funding agency in the public, commercial, or not-for-profit sectors.
